# Crystal structure of 3-meth­oxy­carbonyl-2-(4-meth­oxy­phen­yl)-8-oxo-1-aza­spiro[4.5]deca-1,6,9-trien-1-ium-1-olate

**DOI:** 10.1107/S1600536814023277

**Published:** 2014-10-29

**Authors:** Lucimara Julio Martins, Deborah de Alencar Simoni, Ricardo Aparicio, Fernando Coelho

**Affiliations:** a Laboratory of Synthesis of Natural Products and Drugs, Institute of Chemistry, University of Campinas, PO Box 6154 – 13083-970, Campinas, SP, Brazil; b Laboratory of Single Crystal X-Ray Diffraction, Institute of Chemistry, University of Campinas, PO Box 6154 – 13083-970, Campinas, SP, Brazil; c Laboratory of Structural Biology and Crystallography, Institute of Chemistry, University of Campinas, PO Box 6154 – 13083-970, Campinas, SP, Brazil

**Keywords:** single-crystal X-ray study, spiro­hexa­dienone structure, Morita–Baylis–Hillman aducts

## Abstract

The title compound, C_18_H_17_NO_5_, was prepared by a synthetic strategy based on the Heck reaction from Morita–Baylis–Hillman adducts. The five-membered ring adopts a slightly twisted conformation on the C_a_—C_m_ (a = aromatic and m = methyl­ene) bond. The dihedral angle between the five-membered ring and the spiro aromatic ring is 89.35 (7)°; that between the five-membered ring and the 4-meth­oxy­benzene ring is 4.65 (7)°. Two short intra­molecular C—H⋯O contacts occur. In the crystal, mol­ecules are linked by C—H⋯O hydrogen bonds to generate a three-dimensional network.

## Related literature   

For compounds that contain a spiro­hexa­dienone moiety in their structures, see: Wright & König (1993 ▶); König *et al.* (1990 ▶); Beil *et al.* (1998 ▶) and for their biological activities, see: Glushkov *et al.* (2010 ▶); Pereira *et al.* (2007 ▶). For strategies for the synthesis of spiro-hexa­dienones from Morita–Baylis–Hillman adducts, see: Coelho *et al.* (2002 ▶); Ferreira *et al.* (2009 ▶); Pirovani *et al.* (2009 ▶); Martins *et al.* (2014 ▶). For the biological activity of compounds containing a nitrone group, see: Fangour *et al.* (2009 ▶); Floyd *et al.* (2008 ▶); Halliwell & Gutteridge (1999 ▶); Fevig *et al.* (1996 ▶). For a discussion about non-classical hydrogen bonds, see: Desiraju (2005 ▶).
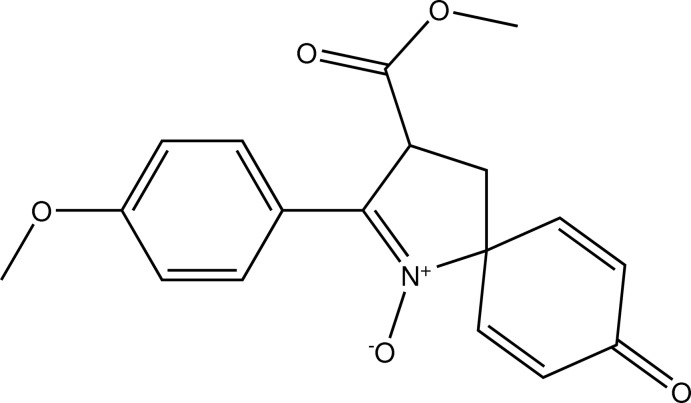


## Experimental   

### Crystal data  

C_18_H_17_NO_5_
*M* *_r_* = 327.32
Triclinic, 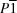

*a* = 6.0916 (11) Å
*b* = 8.7713 (16) Å
*c* = 15.167 (3) Å
α = 80.255 (6)°
β = 81.703 (6)°
γ = 80.122 (6)°
*V* = 781.3 (2) Å^3^
*Z* = 2
Cu *K*α radiation
μ = 0.85 mm^−1^
*T* = 100 K
0.47 × 0.20 × 0.17 mm

### Data collection  

Bruker APEXII CCD diffractometer
Absorption correction: multi-scan (*SADABS*; Bruker, 2010 ▶) *T*_min_ = 0.813, *T*_max_ = 1.000
14656 measured reflections
2771 independent reflections
2727 reflections with *I* > 2σ(*I*)
*R*_int_ = 0.033

### Refinement  

*R*[*F* ^2^ > 2σ(*F* ^2^)] = 0.042
*wR*(*F* ^2^) = 0.105
*S* = 1.11
2771 reflections
220 parameters
H-atom parameters constrained
Δρ_max_ = 0.28 e Å^−3^
Δρ_min_ = −0.35 e Å^−3^

### 

Data collection: *APEX2* (Bruker, 2010 ▶); cell refinement: *SAINT* (Bruker, 2010 ▶); data reduction: *SAINT*; program(s) used to solve structure: *SHELXS97* (Sheldrick, 2008 ▶); program(s) used to refine structure: *SHELXL2014* (Sheldrick, 2008 ▶); molecular graphics: *WinGX* (Farrugia, 2012 ▶) and *PLATON* (Spek, 2009 ▶); software used to prepare material for publication: *publCIF* (Westrip, 2010 ▶).

## Supplementary Material

Crystal structure: contains datablock(s) I, New_Global_Publ_Block. DOI: 10.1107/S1600536814023277/hb7301sup1.cif

Structure factors: contains datablock(s) I. DOI: 10.1107/S1600536814023277/hb7301Isup2.hkl

Supporting information file. DOI: 10.1107/S1600536814023277/hb7301Isup3.cdx

Supporting information file. DOI: 10.1107/S1600536814023277/hb7301Isup4.cml

**Fig. 1.**
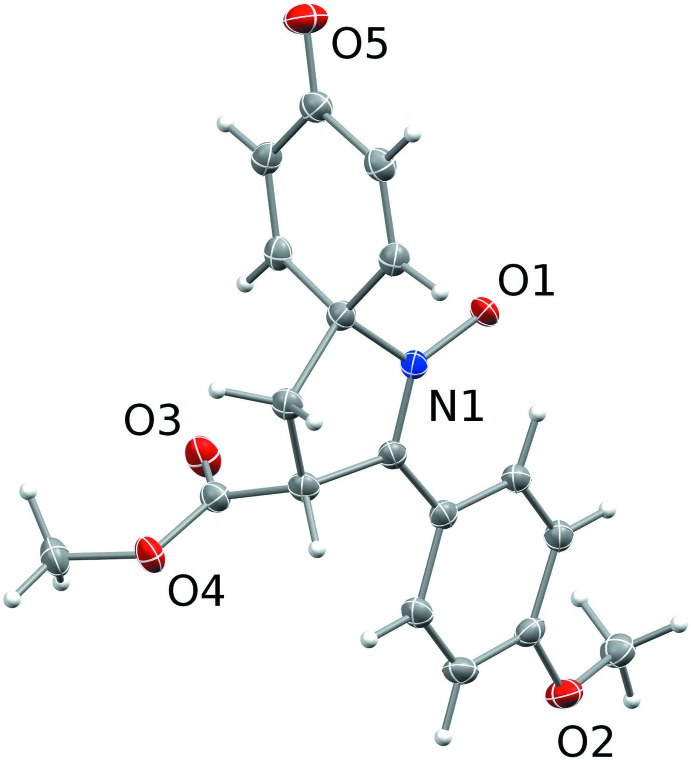
The molecular structure of the title compound with 50% probability displacement ellipsoids.

. DOI: 10.1107/S1600536814023277/hb7301fig1.tif
The mol­ecular structure of the title compound with 50% probability displacement ellipsoids.

**Fig. 2.**
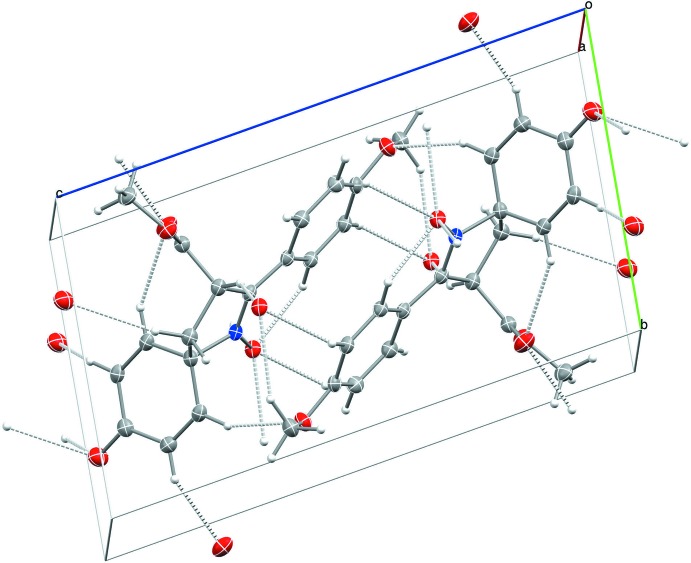
Crystal packing of the title compound, showing hydrogen bonding interactions.

. DOI: 10.1107/S1600536814023277/hb7301fig2.tif
Crystal packing of the title compound, showing hydrogen bonding inter­actions.

CCDC reference: 1030399

Additional supporting information:  crystallographic information; 3D view; checkCIF report

## Figures and Tables

**Table 1:** Hydrogen-bond geometry (, )

*D*H*A*	*D*H	H*A*	*D* *A*	*D*H*A*
C8H1*C*O1^i^	0.98	2.59	3.4941(18)	153
C4H3O1^i^	0.95	2.38	3.1345(16)	136
C3H4O1	0.95	2.22	2.8725(17)	125
C14H8O3	0.95	2.57	3.3431(18)	138
C15H9O5^ii^	0.95	2.56	3.3821(17)	146
C18H13O2^iii^	0.95	2.54	3.4309(17)	155
C17H14O3^iv^	0.95	2.38	3.2408(18)	151
C12H15*A*O5^v^	0.99	2.60	3.5402(18)	159
C9H16O1^vi^	1.00	2.31	3.2045(16)	148

Symmetry codes: (i) 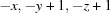; (ii) 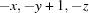; (iii) 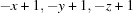; (iv) 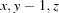; (v) 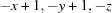; (vi) 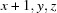.

## supplementary crystallographic information

### S1. Introduction

Natural products, isolated both from terrestrial or marine sources, display great structural diversity and exhibit remarkable biological activities, many of them bearing in their structures a spiro-hexadienone moiety (Wright & König (1993); Beil *et al.* (1998)). Owing to the high conjugation, provided by the presence of a carbonyl group and two double bonds, this structural moiety acts as an efficient Michael acceptor and this chemical property is routinely associated with some biological activities, such as cytotoxic (Pereira *et al.* (2007); Glushkov *et al.* (2010)).

Compounds presenting a nitrone group in their structures can present biological activity related to radical trapping in chemical systems (Fangour *et al.* (2009); Floyd *et al.* (2008); Halliwell *et al.* (1999)). The presence of radicals is normally associated to several type of pathologies. Our inter­est in preparing spiro-hexadienones with great structural diversity combinated with the biological effect that can be associated to nitrone groups stimulated us to synthesize new spiro compounds containing a nitrone group into their structures and evaluate the biological profiles of these new compounds.

A strategy for the synthesis of spiro-hexadienones from Morita-Baylis-Hillman aducts had been developed. This strategy is based on the Heck reaction, followed by phenolic oxidation of functionalized b-ketoester mediated by a hypervalent iodine reagent (Coelho *et al.* (2002); Ferreira *et al.* (2009); Floyd *et al.* (2008); Halliwell *et al.* (1999)). As far as we know, we synthesized for the first time new functionalized aza­spiro compounds from Morita-Baylis-Hillman.

### S2.1. Synthesis and crystallization

Some β-ketoesters, prepared from Morita-Baylis-Hillman adducts, were treated with hydroxyl­amine hydro­chloride to furnish a diastereomeric mixture of oximes, in which the E isomer cyclizes spontaneously to the corresponding isoxazoles. The Z oxime was treated with PIFA [phenyl­iodine(III) bis­(tri­fluoro­acetate)] to furnish the new aza­spiro compounds in moderate overall yield (3-17%). The obtained 3-(Meth­oxy­carbonyl)-2-(4-meth­oxy­phenyl)-8-oxo-1-aza­spiro­[4.5]deca-1,6,9-trien-1-ium-1-olate (33 mg, 0.1 mmol) was dissolved in absolute chloro­form-D1 (1 mL), followed by stirring until total dissolution was achieved. The solution was kept in the freezer. After two weeks, the resulting solution was filtered using a vacuum, washed with small portions of cold chloro­form and dried in a desiccator to furnish colourless prisms.

### S2.2. Refinement

A riding model was used to calculate the positions of included H atoms, with aromatic and methyl C—H bond lengths of 0.95 and 0.98 A °, respectively. The isotropic displacement parameters values (Uiso(H)) were fixed at 1.5Ueq(C) for methyl H atoms and 1.2Ueq(C) for all other attached H atoms.

### Crystal data

**Table:**

C_18_H_17_NO_5_	*Z* = 2
*M**_r_* = 327.32	*F*(000) = 344
Triclinic, *P*1	*D*_x_ = 1.391 Mg m^−^^3^
Hall symbol: -P 1	Cu *K*α radiation, λ = 1.54178 Å
*a* = 6.0916 (11) Å	Cell parameters from 109 reflections
*b* = 8.7713 (16) Å	θ = 9.0–38.4°
*c* = 15.167 (3) Å	µ = 0.85 mm^−^^1^
α = 80.255 (6)°	*T* = 100 K
β = 81.703 (6)°	Prismatic, colourless
γ = 80.122 (6)°	0.47 × 0.20 × 0.17 mm
*V* = 781.3 (2) Å^3^	

### Data collection

**Table:**

Bruker APEXII CCD diffractometer	2771 independent reflections
Radiation source: fine-focus sealed tube	2727 reflections with *I* > 2σ(*I*)
Detector resolution: 8.3333 pixels mm^-1^	*R*_int_ = 0.033
φ and ω scans	θ_max_ = 67.7°, θ_min_ = 3.0°
Absorption correction: multi-scan (*SADABS*; Bruker, 2010)	*h* = −5→7
*T*_min_ = 0.813, *T*_max_ = 1.000	*k* = −10→10
14656 measured reflections	*l* = −18→17

### Refinement

**Table:**

Refinement on *F*^2^	Hydrogen site location: inferred from neighbouring sites
Least-squares matrix: full	H-atom parameters constrained
*R*[*F*^2^ > 2σ(*F*^2^)] = 0.042	*w* = 1/[σ^2^(*F*_o_^2^) + (0.0542*P*)^2^ + 0.3281*P*] where *P* = (*F*_o_^2^ + 2*F*_c_^2^)/3
*wR*(*F*^2^) = 0.105	(Δ/σ)_max_ < 0.001
*S* = 1.11	Δρ_max_ = 0.28 e Å^−^^3^
2771 reflections	Δρ_min_ = −0.35 e Å^−^^3^
220 parameters	Extinction correction: *SHELXL2014* (Sheldrick, 2014), Fc^*^=kFc[1+0.001xFc^2^λ^3^/sin(2θ)]^-1/4^
0 restraints	Extinction coefficient: 0.043 (2)

### Special details

**Table:**

Geometry. All e.s.d.'s (except the e.s.d. in the dihedral angle between two l.s. planes) are estimated using the full covariance matrix. The cell e.s.d.'s are taken into account individually in the estimation of e.s.d.'s in distances, angles and torsion angles; correlations between e.s.d.'s in cell parameters are only used when they are defined by crystal symmetry. An approximate (isotropic) treatment of cell e.s.d.'s is used for estimating e.s.d.'s involving l.s. planes.

### Fractional atomic coordinates and isotropic or equivalent isotropic displacement parameters (Å^2^)

**Table:**

	*x*	*y*	*z*	*U*_iso_*/*U*_eq_	
O2	0.20642 (16)	0.89397 (12)	0.62486 (6)	0.0247 (3)	
O5	0.19082 (18)	0.28804 (12)	0.01392 (7)	0.0309 (3)	
O1	0.19910 (14)	0.44109 (11)	0.32280 (6)	0.0199 (2)	
O4	0.91693 (15)	0.79511 (11)	0.19091 (7)	0.0228 (3)	
O3	0.54105 (16)	0.85337 (11)	0.19863 (7)	0.0281 (3)	
N1	0.38917 (17)	0.48769 (12)	0.29230 (7)	0.0166 (3)	
C8	−0.0287 (2)	0.92550 (17)	0.65513 (10)	0.0252 (3)	
H1A	−0.1106	0.9720	0.6036	0.038*	
H1B	−0.0535	0.9985	0.6991	0.038*	
H1C	−0.0827	0.8276	0.6833	0.038*	
C5	0.2673 (2)	0.80731 (16)	0.55566 (9)	0.0202 (3)	
C4	0.1260 (2)	0.72330 (16)	0.52570 (9)	0.0200 (3)	
H3	−0.0222	0.7203	0.5551	0.024*	
C3	0.2017 (2)	0.64391 (15)	0.45269 (9)	0.0189 (3)	
H4	0.1048	0.5855	0.4333	0.023*	
C2	0.4179 (2)	0.64816 (15)	0.40712 (9)	0.0177 (3)	
C1	0.4981 (2)	0.57613 (15)	0.32644 (9)	0.0173 (3)	
C13	0.4972 (2)	0.44582 (15)	0.20162 (9)	0.0180 (3)	
C14	0.3688 (2)	0.55382 (15)	0.13203 (9)	0.0186 (3)	
H8	0.3569	0.6635	0.1312	0.022*	
C15	0.2712 (2)	0.50353 (16)	0.07177 (9)	0.0202 (3)	
H9	0.1934	0.5777	0.0293	0.024*	
C16	0.2811 (2)	0.33526 (16)	0.06944 (9)	0.0216 (3)	
C7	0.5596 (2)	0.73034 (16)	0.44026 (9)	0.0207 (3)	
H11	0.7083	0.7332	0.4114	0.025*	
C6	0.4867 (2)	0.80683 (16)	0.51396 (9)	0.0223 (3)	
H12	0.5863	0.8593	0.5363	0.027*	
C18	0.4924 (2)	0.27616 (15)	0.20004 (9)	0.0197 (3)	
H13	0.5580	0.2010	0.2454	0.024*	
C17	0.4002 (2)	0.22581 (16)	0.13799 (9)	0.0213 (3)	
H14	0.4117	0.1164	0.1379	0.026*	
C12	0.7367 (2)	0.48121 (15)	0.20051 (9)	0.0196 (3)	
H15A	0.7956	0.5274	0.1394	0.024*	
H15B	0.8389	0.3846	0.2199	0.024*	
C9	0.7139 (2)	0.59949 (15)	0.26787 (9)	0.0186 (3)	
H16	0.8422	0.5732	0.3049	0.022*	
C10	0.7081 (2)	0.76464 (16)	0.21684 (9)	0.0195 (3)	
C11	0.9377 (3)	0.93704 (17)	0.12808 (10)	0.0270 (3)	
H18A	0.8704	0.9329	0.0738	0.040*	
H18B	1.0968	0.9470	0.1118	0.040*	
H18C	0.8601	1.0274	0.1561	0.040*	

### Atomic displacement parameters (Å^2^)

**Table:**

	*U*^11^	*U*^22^	*U*^33^	*U*^12^	*U*^13^	*U*^23^
O2	0.0232 (5)	0.0323 (6)	0.0211 (5)	−0.0051 (4)	−0.0046 (4)	−0.0084 (4)
O5	0.0324 (6)	0.0323 (6)	0.0319 (6)	−0.0051 (5)	−0.0150 (5)	−0.0066 (4)
O1	0.0130 (5)	0.0267 (5)	0.0208 (5)	−0.0079 (4)	−0.0007 (4)	−0.0017 (4)
O4	0.0166 (5)	0.0233 (5)	0.0272 (5)	−0.0063 (4)	0.0016 (4)	−0.0003 (4)
O3	0.0188 (5)	0.0246 (5)	0.0372 (6)	−0.0016 (4)	−0.0037 (4)	0.0044 (4)
N1	0.0128 (5)	0.0199 (5)	0.0161 (5)	−0.0027 (4)	−0.0024 (4)	0.0005 (4)
C8	0.0252 (7)	0.0298 (7)	0.0210 (7)	−0.0038 (6)	−0.0018 (6)	−0.0061 (6)
C5	0.0233 (7)	0.0216 (7)	0.0158 (6)	−0.0023 (5)	−0.0067 (5)	−0.0005 (5)
C4	0.0179 (6)	0.0231 (7)	0.0183 (6)	−0.0047 (5)	−0.0025 (5)	0.0008 (5)
C3	0.0180 (6)	0.0209 (6)	0.0184 (6)	−0.0057 (5)	−0.0048 (5)	0.0004 (5)
C2	0.0163 (6)	0.0187 (6)	0.0174 (6)	−0.0028 (5)	−0.0051 (5)	0.0022 (5)
C1	0.0143 (6)	0.0185 (6)	0.0182 (6)	−0.0023 (5)	−0.0051 (5)	0.0019 (5)
C13	0.0139 (6)	0.0229 (7)	0.0166 (6)	−0.0016 (5)	−0.0014 (5)	−0.0023 (5)
C14	0.0140 (6)	0.0202 (6)	0.0190 (6)	−0.0005 (5)	0.0006 (5)	−0.0004 (5)
C15	0.0148 (6)	0.0250 (7)	0.0184 (6)	−0.0002 (5)	−0.0023 (5)	0.0011 (5)
C16	0.0156 (6)	0.0280 (7)	0.0214 (7)	−0.0036 (5)	−0.0017 (5)	−0.0039 (6)
C7	0.0153 (6)	0.0238 (7)	0.0229 (7)	−0.0043 (5)	−0.0045 (5)	−0.0001 (5)
C6	0.0199 (7)	0.0245 (7)	0.0246 (7)	−0.0054 (5)	−0.0094 (5)	−0.0019 (5)
C18	0.0159 (6)	0.0216 (7)	0.0195 (6)	0.0000 (5)	−0.0017 (5)	0.0000 (5)
C17	0.0180 (7)	0.0208 (7)	0.0243 (7)	−0.0027 (5)	−0.0017 (5)	−0.0021 (5)
C12	0.0136 (6)	0.0226 (7)	0.0219 (7)	−0.0013 (5)	−0.0027 (5)	−0.0017 (5)
C9	0.0130 (6)	0.0218 (7)	0.0204 (6)	−0.0021 (5)	−0.0037 (5)	−0.0006 (5)
C10	0.0159 (6)	0.0231 (7)	0.0197 (7)	−0.0041 (5)	−0.0013 (5)	−0.0033 (5)
C11	0.0275 (8)	0.0252 (7)	0.0277 (7)	−0.0109 (6)	0.0023 (6)	0.0002 (6)

### Geometric parameters (Å, º)

**Table:**

O2—C5	1.3705 (17)	C13—C14	1.5066 (18)
O2—C8	1.4339 (17)	C13—C12	1.5395 (17)
O5—C16	1.2301 (17)	C14—C15	1.3289 (19)
O1—N1	1.2905 (14)	C14—H8	0.9500
O4—C10	1.3345 (16)	C15—C16	1.473 (2)
O4—C11	1.4462 (17)	C15—H9	0.9500
O3—C10	1.2059 (17)	C16—C17	1.4732 (19)
N1—C1	1.3121 (17)	C7—C6	1.380 (2)
N1—C13	1.5121 (16)	C7—H11	0.9500
C8—H1A	0.9800	C6—H12	0.9500
C8—H1B	0.9800	C18—C17	1.332 (2)
C8—H1C	0.9800	C18—H13	0.9500
C5—C4	1.3908 (19)	C17—H14	0.9500
C5—C6	1.393 (2)	C12—C9	1.5519 (18)
C4—C3	1.3884 (19)	C12—H15A	0.9900
C4—H3	0.9500	C12—H15B	0.9900
C3—C2	1.3996 (19)	C9—C10	1.5197 (18)
C3—H4	0.9500	C9—H16	1.0000
C2—C7	1.4067 (18)	C11—H18A	0.9800
C2—C1	1.4554 (19)	C11—H18B	0.9800
C1—C9	1.5014 (18)	C11—H18C	0.9800
C13—C18	1.4977 (19)		
			
C5—O2—C8	116.78 (10)	C16—C15—H9	119.3
C10—O4—C11	115.74 (11)	O5—C16—C15	121.67 (13)
O1—N1—C1	128.90 (11)	O5—C16—C17	121.42 (13)
O1—N1—C13	116.85 (10)	C15—C16—C17	116.89 (12)
C1—N1—C13	114.09 (10)	C6—C7—C2	121.25 (12)
O2—C8—H1A	109.5	C6—C7—H11	119.4
O2—C8—H1B	109.5	C2—C7—H11	119.4
H1A—C8—H1B	109.5	C7—C6—C5	120.13 (12)
O2—C8—H1C	109.5	C7—C6—H12	119.9
H1A—C8—H1C	109.5	C5—C6—H12	119.9
H1B—C8—H1C	109.5	C17—C18—C13	123.01 (12)
O2—C5—C4	124.47 (12)	C17—C18—H13	118.5
O2—C5—C6	115.89 (12)	C13—C18—H13	118.5
C4—C5—C6	119.64 (12)	C18—C17—C16	121.73 (13)
C3—C4—C5	119.90 (12)	C18—C17—H14	119.1
C3—C4—H3	120.1	C16—C17—H14	119.1
C5—C4—H3	120.1	C13—C12—C9	105.07 (10)
C4—C3—C2	121.34 (12)	C13—C12—H15A	110.7
C4—C3—H4	119.3	C9—C12—H15A	110.7
C2—C3—H4	119.3	C13—C12—H15B	110.7
C3—C2—C7	117.62 (12)	C9—C12—H15B	110.7
C3—C2—C1	123.04 (12)	H15A—C12—H15B	108.8
C7—C2—C1	119.31 (12)	C1—C9—C10	112.58 (11)
N1—C1—C2	125.30 (12)	C1—C9—C12	103.83 (10)
N1—C1—C9	110.32 (11)	C10—C9—C12	109.86 (11)
C2—C1—C9	124.32 (11)	C1—C9—H16	110.1
C18—C13—C14	113.42 (11)	C10—C9—H16	110.1
C18—C13—N1	109.22 (10)	C12—C9—H16	110.1
C14—C13—N1	106.44 (10)	O3—C10—O4	124.52 (12)
C18—C13—C12	112.45 (11)	O3—C10—C9	125.53 (12)
C14—C13—C12	113.17 (11)	O4—C10—C9	109.87 (11)
N1—C13—C12	101.15 (10)	O4—C11—H18A	109.5
C15—C14—C13	123.35 (12)	O4—C11—H18B	109.5
C15—C14—H8	118.3	H18A—C11—H18B	109.5
C13—C14—H8	118.3	O4—C11—H18C	109.5
C14—C15—C16	121.39 (12)	H18A—C11—H18C	109.5
C14—C15—H9	119.3	H18B—C11—H18C	109.5

### Hydrogen-bond geometry (Å, º)

**Table:**

*D*—H···*A*	*D*—H	H···*A*	*D*···*A*	*D*—H···*A*
C8—H1*C*···O1^i^	0.98	2.59	3.4941 (18)	153
C4—H3···O1^i^	0.95	2.38	3.1345 (16)	136
C3—H4···O1	0.95	2.22	2.8725 (17)	125
C14—H8···O3	0.95	2.57	3.3431 (18)	138
C15—H9···O5^ii^	0.95	2.56	3.3821 (17)	146
C18—H13···O2^iii^	0.95	2.54	3.4309 (17)	155
C17—H14···O3^iv^	0.95	2.38	3.2408 (18)	151
C12—H15*A*···O5^v^	0.99	2.60	3.5402 (18)	159
C9—H16···O1^vi^	1.00	2.31	3.2045 (16)	148

Symmetry codes: (i) −*x*, −*y*+1, −*z*+1; (ii) −*x*, −*y*+1, −*z*; (iii) −*x*+1, −*y*+1, −*z*+1; (iv) *x*, *y*−1, *z*; (v) −*x*+1, −*y*+1, −*z*; (vi) *x*+1, *y*, *z*.
