# Supplementary figures and images for: Crystal structure of 3-meth­oxy­carbonyl-2-(4-meth­oxy­phen­yl)-8-oxo-1-aza­spiro[4.5]deca-1,6,9-trien-1-ium-1-olate
Source: Acta Crystallogr Sect E Struct Rep Online. 2014 Oct 29;70(Pt 11):o1200–1. doi: 10.1107/S1600536814023277 (PMC4257292; doi:10.1107/S1600536814023277)

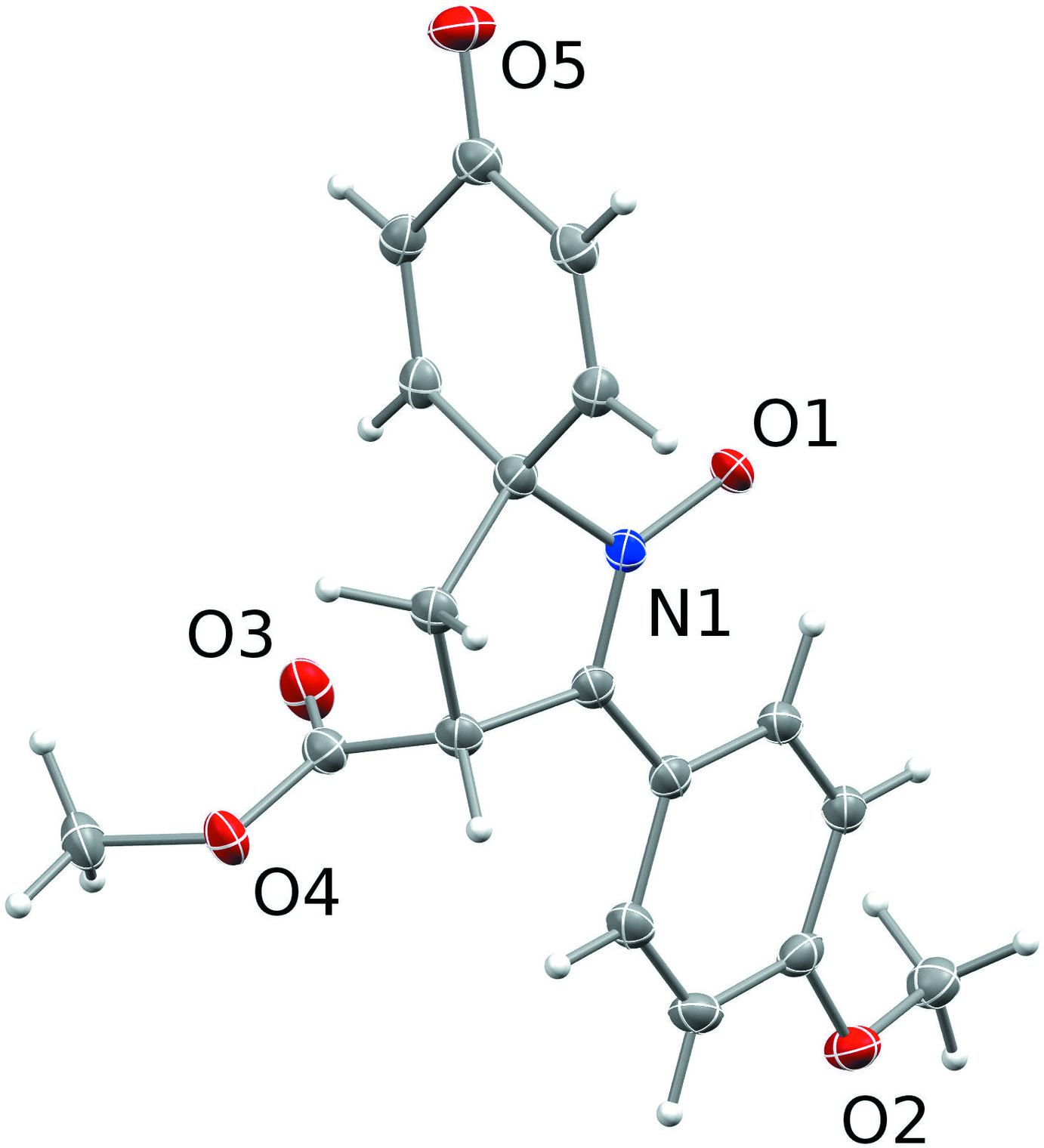

Supplement: Supplementary file 5 [file e-70-o1200-fig1.tif]

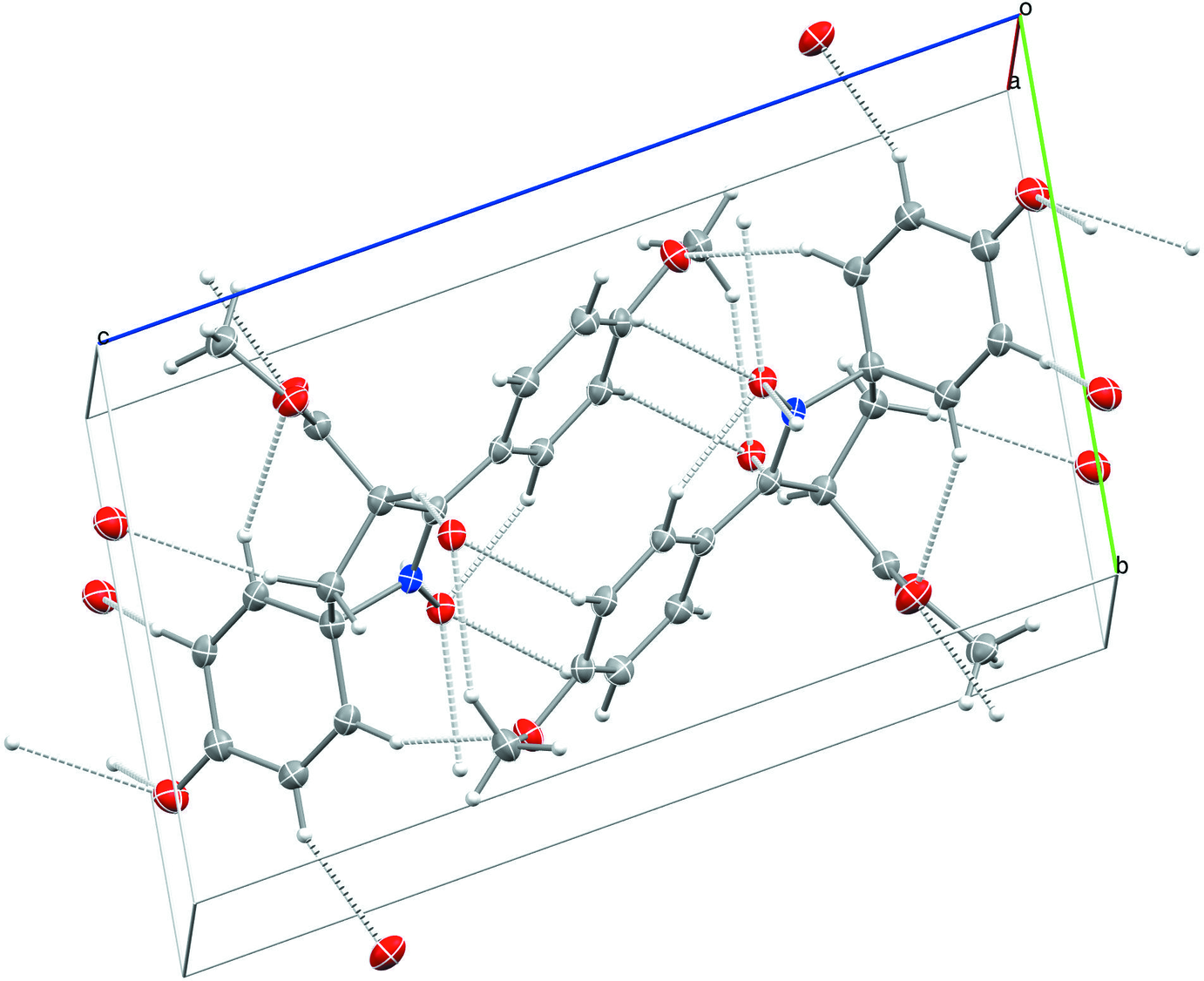

Supplement: Supplementary file 6 [file e-70-o1200-fig2.tif]
